# Place-based household vouchers for locally supplied fruit and vegetables: the Fresh Street pilot cluster randomised controlled trial

**DOI:** 10.1186/s12889-024-21062-y

**Published:** 2025-01-03

**Authors:** C. Relton, M. K. Blake, G. Bridge, D. Umney, S. J. C. Taylor, J. Adams, B. Mihaylova, C. Griffiths, R. Hooper, R. Phillips, L. Palmer, A. Gamston, K. Williamson

**Affiliations:** 1 Wolfson Institute of Population Health, Queen Mary University of London, London, E1 2AB UK; 2 Department of Geography, University of Sheffield, Sheffield, S10 2TN UK; 3 School of Geography, University of Leeds, Woodhouse, Leeds, LS2 9JT UK; 4 MRC Epidemiology Unit, University of Cambridge, Cambridge, CB2 0SL UK; 5 Nuffield Department of Population Health, University of Oxford, Oxford, OX3 7LF UK; 6 Grounded Research Hub, Rotherham Doncaster and South Humber NHS Foundation Trust, Doncaster, DN4 8QN UK; 7 School of Public Health, Imperial College, London, SW7, 2BX UK

**Keywords:** Fruit and vegetables, Place-based approach, Food deserts, Deprivation, Household vouchers, Pilot randomised controlled trial

## Abstract

**Background:**

Households in areas of socio-economic deprivation are more likely to consume diets low in fruit and vegetables. Fresh Street is a place-based fruit and vegetable voucher scheme with vouchers redeemable with local independent (non-supermarket) vendors. Paper vouchers are offered to all households in a geographical area regardless of household type, size, or income with no requirement to demonstrate need. The regular shareable vouchers are combined with recipes and dietary information to increase exposure to healthy food prompts, reduce food insecurity, increase fruit and vegetable consumption, improve dietary quality, and support healthy dietary habits.

This study aimed to inform a randomised controlled trial (RCT) to assess the impact of Fresh Street on a range of public health outcomes.

**Methods:**

The pilot cluster RCT took place in three inner city areas of high socioeconomic deprivation in England (Tower Hamlets, Bradford, and Doncaster). New systems for managing vouchers and doorstep delivering weekly envelopes to households were developed. Weekly envelopes containing vouchers (5 x £1), a healthy seasonal recipe and brief nutritional information were offered to all households in nine intervention streets. Nine control streets received no intervention. Household surveys collected information on fruit and vegetable consumption, diet quality, and household characteristics.

**Results:**

The household survey response rate was below the 50% target for progression to the main trial. Most local fruit and vegetable vendors accepted vouchers. Three quarters or more of households regularly accepted the envelopes. The scheme was well received by households, local vendors and local public health teams. Household uptake of the scheme was highest in Tower Hamlets (75%) and Bradford (83%). The mean weekly voucher redemption was highest in Tower Hamlets (£3.26) and Bradford (£2.82), where the scheme ran longest, and where vendors were nearby.

**Conclusions:**

This was the first pilot RCT of a place-based, household voucher approach. The newly developed system for securely printing and redeeming the vouchers worked well and is potentially scalable.

Future trials should consider alternative methods of assessing the impact on households and explore more efficient ways to deliver the intervention e.g. through collaborative working with local resources such as community centres.

**Supplementary Information:**

The online version contains supplementary material available at 10.1186/s12889-024-21062-y.

## Background

The diets of many UK populations do not meet government guidelines [1]. Food prices are a key, but not the only, determinant of food consumption [1]. People living in areas of high socio-economic deprivation are more likely to consume diets higher in sugar and saturated fats, and lower in fruit and vegetables and dietary fibre [2] and report price as a barrier to healthy eating [3]. They are also more likely to experience food insecurity [4]: *‘the state of being without consistent and reliable access to a sufficient quantity of affordable, nutritious food’* [5] and live in food deserts where there is limited access to fruit and vegetables [6]. These factors, in combination, contribute to sub-optimal fruit and vegetable consumption and increased preventable morbidity and mortality [7, 8].

Offering price discounts or subsidies on healthy foods increases healthy food purchasing [9] and consumption [10], especially when access to fruit and vegetables is also improved [11]. Other interventions such as nutrition education can help people know what foods are healthy, and how to incorporate them into their diets [12].

Targeted voucher or cash transfer programmes can support healthier diets. However, individually targeted programmes have limitations. The UK ‘Healthy Start’ programme offers pregnant women and carers of children under four living in households on some benefits prepaid cards for fruit and vegetables, pulses, milk, and infant formula [13]. Although observational data suggests ‘Healthy Start’ increases fruit and vegetable intake, 37% of those eligible do not use the scheme [14] and concerns exist regarding the stigma associated with its targeted nature [15] and the fact that most Healthy Start vouchersF for children under 1 year are used for infant formula [16]. There is a need for effective and cost-effective interventions that increase fruit and vegetable intake and support a shift towards healthier diets in the UK, particularly for those living in areas of high deprivation.

Fresh Street is a place-based fruit and vegetable voucher scheme with no requirement for individuals to demonstrate need. Vouchers are offered to all households in a geographical area regardless of household type, size, or income. Weekly shareable vouchers are combined with regular recipes and dietary information to increase exposure to healthy food prompts, reduce food insecurity, increase fruit and vegetable consumption, improve dietary quality, and support healthy dietary habits [17] (see Fresh Street Theory of Change supplementary material 1). To increase the resilience of the local food system, vouchers are redeemable only with local, independent fruit and vegetable vendors (not supermarkets).

The Fresh Street intervention was initially developed in consultation with households and one local Public Health team, and feasibility tested in two areas of high deprivation in the north of England using rapid ethnographic assessment [18, 19]. The first site (Barnsley) included 4 streets (97 households) and the second site in Sheffield was a block of 54 two-bedroom flats. In both locations vouchers (5 x £1) plus recipes and dietary information were delivered every week to participating households for one year. Vouchers were redeemable at fruit and vegetable stalls in city centre markets approximately 3 miles away. In Barnsley there was also a local independent fruit and vegetable shop ~ 0.3 miles away. In Sheffield households could opt to receive a delivered prepacked £5 mixed bag of fruit and vegetables. More than three quarters of all eligible households used the voucher scheme [17, 19]. Householders reported that the scheme made them think more about what they were eating and prompted them to buy and eat more fruit and vegetables. During doorstep conversations householders frequently talked unprompted about their health. Local fruit and vegetable vendors reported new customers as well as existing customers buying more. Interest in the delivered prepacked bag was low with most households preferring to travel to the market stalls to choose their fruit and vegetables [19]. Whilst the feasibility study indicated high levels of acceptance and engagement with the intervention, its impact on diet and health markers has not yet been quantified or evaluated in a randomised controlled trial, nor has an efficient scalable system for delivering the intervention been developed. The aim of this pilot study was to inform the design and conduct of a definitive trial to evaluate the impact of the Fresh Street intervention on a range of diet and health outcomes.

## Methods

The objectives were to: 1. Demonstrate baseline primary outcome measure responses were obtainable from at least 50% of households, 2. Develop and deliver intervention in different sites, 3. Assess uptake of the intervention and identify factors which may impact on intervention success, 4. Gain insight into the perspectives of key stakeholder groups, 5. Secure intervention funding for at least 856 households for at least two years for the main trial, and 6. Collect outcome data from pilot sites if the pilot did not progress to the main trial stage.

This study was a parallel group, pilot cluster randomized controlled trial (RCT) with an integrated process evaluation. We report this study using CONSORT extension to randomised pilot and feasibility trials [20].

**Fig. 1:**
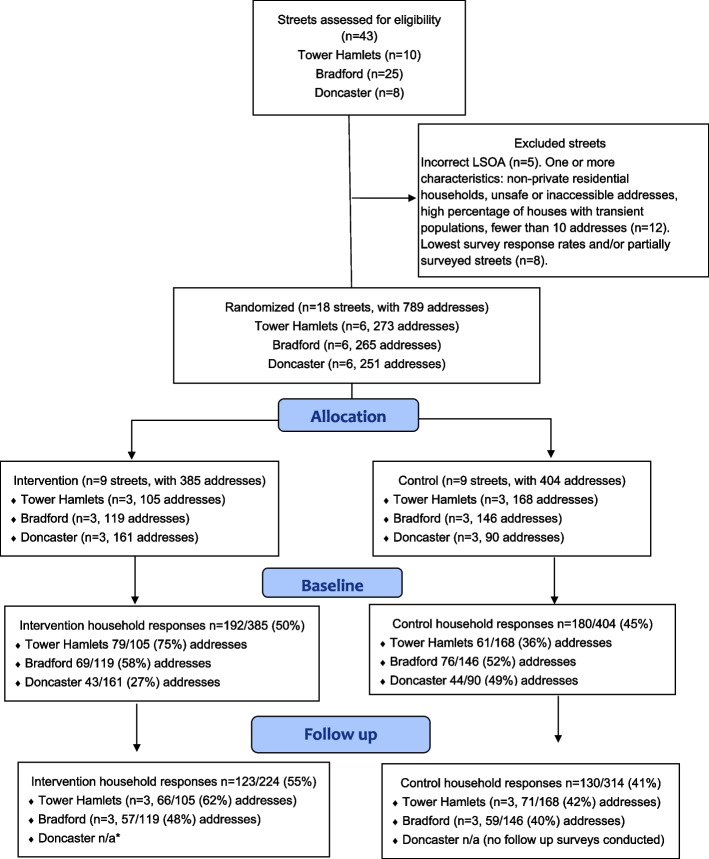
Cluster recruitment and follow-up according to the CONSORT 2010 flow diagram

The study took place in three local government areas in England: London Borough of Tower Hamlets Council, Bradford Metropolitan Borough Council, and Doncaster Metropolitan Borough Council, with support and overarching permission to conduct the study obtained from area local councillors, local councils, and, in Doncaster, the local NHS Trust. The sampling frame for the trial consisted of geographical areas of high deprivation. In consultation with local government public health teams 43 streets were selected based on deprivation levels and availability of local independent fruit and vegetable vendors (Fig. 1). After exclusions 18 streets were randomised. Randomisation of streets was carried out by an independent statistician at Queen Mary University of London Pragmatic Clinical Trials Unit after baseline surveys, with a 1:1 allocation ratio between intervention and control groups. Stratified (by site) permuted blocked randomisation with block sizes of m = 6 and 4 was used to ensure a similar number of clusters within each arm.

### Intervention

**Fig. 2:**
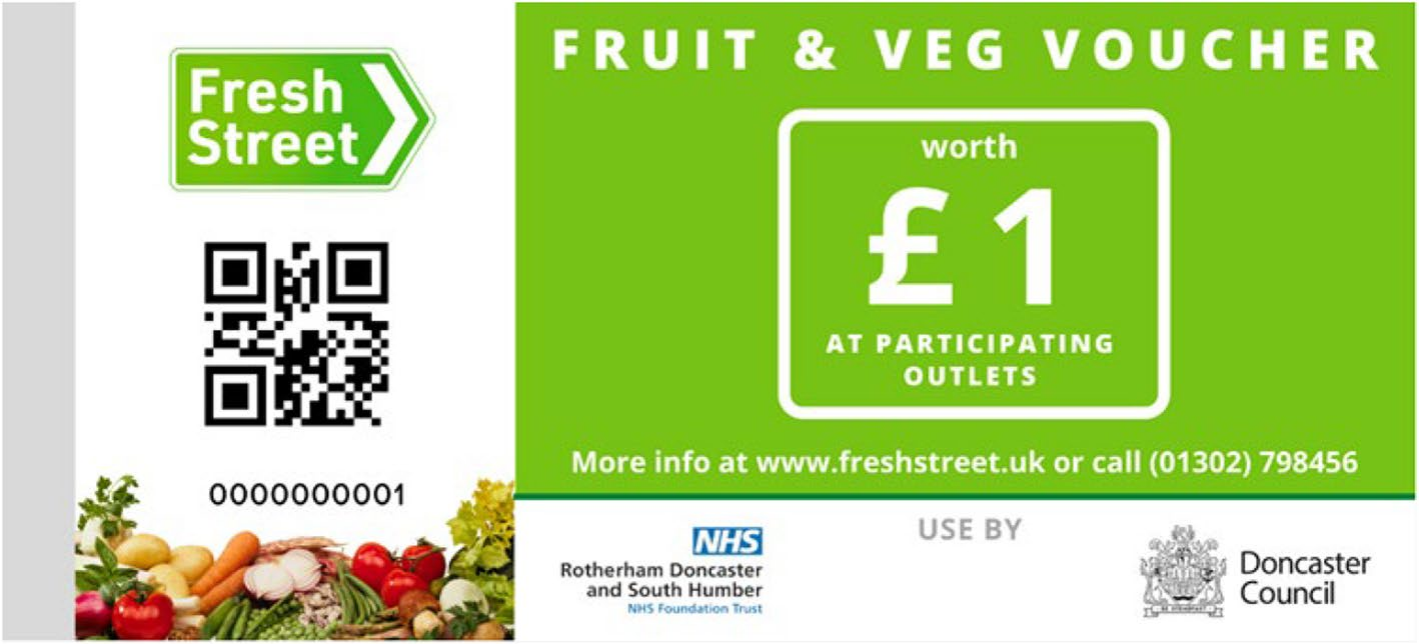
Fresh Street Voucher

The weekly envelopes for households contained 5 x £1 vouchers for locally supplied fruit and vegetables (Fig. 2) redeemable only with local independent vendors, plus weekly letters with recipes and simple brief nutritional information (Supplementary material 3). The intervention is fully described in Table 1 using TIDIeR guidelines [21]. The control group received no intervention.

**Table 1:** Description of the intervention

**Brief name**	**Fresh Street **
**Why:** *Rationale*	The combination of fiscal measures with regular multi-faceted communication/ marketing information is designed to increase consumption of fresh fruit and vegetables, improve diet quality, reduce food insecurity, increase exposure to healthy food prompts and help re-orientate local food systems to advance the economic, social and environmental goals which impact health. Vouchers are paper (not e-vouchers) to ensure they can be used by anyone regardless of digital literacy and resources, and to facilitate sharing.
**Who**: *Recipients of the intervention*	All residential households in the target area. No requirement for households to demonstrate need.
**What**: *Physical or informational materials used in the intervention*	Weekly envelopes delivered by hand to individual households with five x £1 branded vouchers printed with a 6-week expiry date plus: - A healthy, seasonal vegetable-based recipe and related simple nutrition and health/diet information developed by the research team - Details (location etc.) of fruit and vegetable vendors - Additional relevant material from local government public health initiatives Vouchers are redeemable only with local independent fruit and vegetable vendors (not supermarkets). Vendors had to have at least 80% of their total stock as fruit and vegetables to be eligible.
**What**: *Procedures, activities and/or processed used on the intervention*	Printing of letters and secure, traceable vouchers. Vouchers were numbered. Voucher numbers inserted into weekly envelope recorded for each household (to enable households not using vouchers to be identified) Brief training of fruit and vegetable vendors (what items vouchers can be used for and how to redeem vouchers) Weekly letters with healthy vegetable-based recipes and simple brief nutritional information and advice. Households spend vouchers with vendors. Retailers scan vouchers with the voucher reimbursement app. Vendors reimbursed for the voucher value.
**Who**: *intervention providers/ implementers*	Teams write and print envelope materials. Local partners deliver envelopes. Local vendors sell households fruit and vegetables in return for vouchers. Sites facilitate weekly delivery of envelopes
**How**: *Mode of delivery*	Envelopes packed and delivered by community partners or local research staff.
**Where**: *Location of the intervention*	All households in participating streets. Fresh fruit and vegetables supplied by local market traders at their premises or through their existing delivery channels.
**When and how much**: *Duration and dosage of the intervention*	Households receive the intervention (5 x £1 vouchers) every week. To compensate for the one-week Christmas break, households received double vouchers the week before.
**Tailoring**: *Adaptation of the intervention*	Recipes and healthy eating messages tailored to each setting (e.g., dietary habits, ethnic profiles, food geographies, and local priorities and resources). In Tower Hamlets the majority of residents were of Bangladeshi origin, so popular Bangladeshi recipes and the South Asian version of the Eat Well guide for households were used.
**How well (planned):** *Strategies to maximise effective implementation*	Explore the extent of vouchers used to purchase non-fruit and vegetable items using ‘mystery shoppers’. Vouchers have unique QR codes (thus secure and traceable) providing a fully auditable system.
**How well (actual):** *Evidence of implementation variability*	The newly developed secure mobile app-based scanning and voucher monitoring system and systems for delivering envelopes to households were effective. Further refinements needed to improve efficiency.

As part of the intervention a new system for securely printing and managing vouchers was developed with a UK security printing specialist organisation (https://www.a1securityprint.com/).

In order to pack and deliver envelopes to households and reimburse fruit and vegetable vendors, new systems were developed using resources provided by public health teams and research staff at each site. Occasionally, written materials related to healthy eating [22, 23], poverty [24, 25], and weight management [26] were added to the weekly envelopes by public health teams in Tower Hamlets and Bradford.

Local independent fruit and vegetable vendors were approached and asked if they would accept the vouchers. Every voucher issued had a 6 week (42 day) expiry date stamp to encourage regular purchasing and consumption of fruit and vegetables by households and discourage stockpiling or bulk trading. However, vendors were told to accept all voucher regardless of the expiry date in order to support smooth interactions between vendors and households. Local vendors scanned the vouchers using the newly developed mobile-friendly app.

To minimise waste, envelope delivery to a household was stopped if none of their vouchers had ever been scanned at several checkpoints during the intervention delivery period. This checkpoint was conducted at two of the three sites (Tower Hamlets weeks 9 and 26, Bradford week 10) (Supplementary material 4). Households and vendors were notified four weeks before the last voucher delivery that the scheme would end. Vouchers could be used for an additional six weeks after the last voucher delivery date.

All vendors were locally owned businesses, mostly indoor or outdoor market stalls open six days a week (Table 4). The distance between streets and vendors varied. In Doncaster and Bradford, the fruit and vegetable stalls were in the town centre 2.5–3.0 miles away, but in Tower Hamlets the outdoor fruit and vegetable market stalls were closer (~ 0.5 miles). In Bradford, as well as two town centre market stalls a local community centre shop sold some fruit and vegetables, but these were low in quality and quantity. From week 6 onwards a local fruit and vegetable van began weekly visits to the streets.

### Inclusion and exclusion criteria

The aim was to identify a small number of residential streets with relatively stable populations. To achieve this research teams visited each of the 43 selected streets and household surveys initiated. During this process the following streets were excluded: incorrect area (*n* = 5), streets with any of the following characteristics: non-private residential households, unsafe or inaccessible addresses, high percentage of houses with transient populations, fewer than 10 addresses (*n* = 12) (Fig. 1).

### Assessment

Each eligible household was visited by the survey team and one adult invited to complete an area branded “Food and Health” survey and offered a £5 Tesco Shopping Voucher for survey completion. Completed surveys were returned to the survey team or posted. The survey instrument was developed iteratively using different survey lengths and levels of requests for personal demographic information in the first site (Tower Hamlets). This resulted in questions that households were less willing to answer (food insecurity, health-related quality of life, COVID diagnosis and vaccine status) being removed to create a shorter (4 sides of A4) version which was then used in the second and third sites (Bradford and Doncaster) (Supplementary material 2). All survey versions collected information on portions of fruit and vegetables eaten yesterday (primary outcome for the planned main trial) using two questions from the annual Active Lives Survey [27], diet quality [28], life satisfaction, COVID symptoms, long term health conditions (Adapted from [29, 30]), health-service use (Adapted from [29]) and demographic information on ethnicity, number, and age of other household members.

Baseline surveys were conducted prior to randomisation. Follow-up surveys were conducted at two sites after randomisation (Tower Hamlets at 31 weeks and Bradford at 32 weeks). No follow-up survey was conducted at the third site (Doncaster) due to early closure of the pilot study.

Process data on the intervention (delivery, voucher uptake, and redemption) were collected throughout the intervention delivery period. Mystery shoppers [31] at each site visited the fruit and vegetable vendors to assess the potential exchange of vouchers for non-fruit and vegetable items. To gain insight into key stakeholder perspectives, field notes were collected during conversations with households, local fruit and vegetable vendors, and public health teams. The two progression criteria agreed to help decide if it was feasible to proceed to the main trial were a 50% response rate to the household survey and the study obtaining intervention funding for the intervention group (856 households) for the main trial.

### Statistics/ analysis section

As a pilot study, no statistical significance testing was performed, and the focus was on data description, with survey response rates, intervention delivery, uptake of the scheme, voucher use, and outcome data reported for each site and the control and intervention groups. We report the vendor type and distance from streets, duration of the intervention (weeks), weekly household participation rate (% of eligible households accepting the weekly envelopes and having at least one voucher scanned at the checkpoints), proportion of vouchers distributed redeemed, and the average weekly voucher spend of households receiving weekly envelopes and of all eligible households (Table 4).

Key stakeholder perspectives were summarized based on field and meeting notes. Surveys returned without address information were excluded. Due to the nature of the intervention blinding was not possible at any level.

## Results

The pilot study was conducted over 13 months (October 17, 2021, to October 19, 2022) across three sites with 18 streets and 789 household addresses.

### Survey responses

Across the three sites a total of 43 streets were assessed, of which 17 were ineligible. Prior to randomisation further streets (*n* = 8) were excluded in order to not exceed the data collection and intervention resources available for each site. These were those streets (partly or fully surveyed) with the lowest response rates. Almost all (7/8) were in Bradford where the local survey team attempted to survey all 20 streets in the Lower Super Output Area.

A total of 18 eligible streets were randomised (Fig. 1). At baseline, data was sought from 789 addresses of which 17 were reported as unoccupied. Baseline responses were obtained from 372/772 (48.2%) households approached. Baseline survey response rates varied by site (Tower Hamlets 52.6%, Bradford 54.7%, Doncaster 36.4%) and the mean response rate (47.1%) was below the 50% progression criteria. Baseline household survey respondents were more likely to be female, and age and ethnicity varied across the sites with a higher proportion of 18–34-year-olds in Bradford than in Tower Hamlets and Doncaster (Table 2). Survey respondents predominantly self-identified with White ethnic categories (White British and White Other) in Bradford and Doncaster and predominantly Asian categories (Asian British and Asian) respondents in Tower Hamlets. Overall characteristics of respondents in intervention and control sites were very similar (Table 2). Follow-up surveys were sought in two of the three sites and responses obtained from 253/538 (47.0%) households approached (Fig. 1).

**Table 2:** Demographics of the baseline survey respondents by site and group allocation

**Site**	**Demographic**	**Level**	**Intervention**		**Control**	
			**(N)**	**(%)**	**(N)**	**(%)**
Tower Hamlets	Age group (year)	18–34	17	21.5	15	24.6
(*N* = 140)		35 + ^a^	39	49.4	25	41.0
		No data	23	29.1	21	34.4
	Gender	Male	25	31.6	14	23.0
		Female	36	45.6	26	42.6
		No data	18	22.8	21	34.4
	Ethnic group	Asian	42	53.2	32	52.5
		All other^b^	17	21.5	8	13.1
		No data	20	25.3	21	34.4
	Total		79	100	61	100
Bradford	Age group (year)	18–34	28	40.6	24	31.6
(*N* = 145)		35–64	34	49.3	37	48.7
		65 +	7	10.1	14	18.4
		No data	0	0	1	1.3
	Gender	Male	29	42.0	27	35.5
		Female	39	56.5	48	63.2
		No data	1	1.4	1	1.3
	Ethnic group	White	51	73.9	62	81.6
		Asian	9	13.0	< 5	< 6.6
		Other	8	11.6	9	11.8
		No data	1	1.4	< 5	< 6.6
	Total		69	100	76	100
Doncaster	Age group (year)	18–34	6	14.0	6	13.6
(*N* = 87)		35–64	20	46.5	22	50.0
		65 +	17	39.5	16	36.4
		No data	0	0	0	0
	Gender	Male	14	32.6	14	31.8
		Female	29	67.4	30	68.2
		No data	0	0	0	0
	Ethnic group	White	41	95.3	40	90.9
		All other^b^	2	4.7	4	9.1
		No data	0	0	0	0
	Total		43	100	44	100

^a^Age groups 35–64 and 65 + combined to protect small cells

^b^Ethnicity data—modelled on the 2011 Census form—all non-dominant ethnic groups combined to protect small cells

Table 3 reports the mean daily fruit and vegetable consumption (SD) and numbers of participants with and without data (item non-response) by site and group. At baseline 351 respondents provided data on the planned primary outcome for the main trial (fruit and vegetable consumption) and 245 respondents at follow-up with self-reported fruit and vegetable portions per day ranging from 3.70 (SD 2.00) to 4.24 (2.92) at baseline and from 4.31 (2.32) to 4.69 (2.34) at follow-up.

**Table 3:** Fruit and vegetable consumption (portions/day) by site and study group

**Site**	**Group**	**Baseline**			**Follow-up**		
		**Participants with data (N)**	**Participants without data (N)**	**Mean (SD)**	**Participants with data (N)**	**Participants without data (N)**	**Mean (SD)**
Tower Hamlets	Control	57	4	4.04 (2.46)	68	3	4.31 (2.32)
	Intervention	68	11	4.01 (2.30)	64	2	4.69 (2.34)
Bradford	Control	74	2	4.24 (2.92)	58	1	4.67 (2.75)
	Intervention	67	2	3.96 (2.92)	55	2	4.49 (3.00)
Doncaster	Control	44	0	3.70 (2.00)	N/A	N/A	N/A
	Intervention	41	2	3.78 (2.67)	N/A	N/A	N/A

#### Intervention delivery and scheme uptake

Weekly envelopes with vouchers and recipes were delivered to all households (*n* = 375) in the nine intervention streets. The duration of the intervention delivery period varied by site due to study delays; 43 weeks in Tower Hamlets (16.11.21 –07.9.22), 40 weeks in Bradford (09.12.21—08.9.22) and 19 weeks in Doncaster (27.4.22—02.9.22). Almost all householders accepted the envelopes at the start of the scheme. By the time households were notified that the scheme was ending most households in Tower Hamlets (75%) and Bradford (83%) were receiving envelopes and had used vouchers. In Doncaster 95% of households were receiving envelopes but as there was no checkpoint for their use, it is likely some households were receiving envelopes but had never used any vouchers.

#### Voucher redemption

Of all eligible (*n* = 385) households, Tower Hamlets (£2.44) and Bradford (£2.35) had the highest average weekly voucher spend and Doncaster (£1.39) the lowest. Of households receiving weekly envelopes, voucher redemption levels were again highest in Tower Hamlets (84%) and Bradford (67%) and lowest in Doncaster (54%) with a similar pattern seen for average weekly voucher spend: Tower Hamlets (£3.26), Bradford (£2.82) and Doncaster (£1.47) (Table 4). Voucher redemption levels were also higher in sites where the scheme had been running longer and in sites where vendors were nearby e.g. the distance to the Tower Hamlets fruit and vegetable market stalls was ~ 0.5 miles. In Bradford voucher redemption levels were low when households could only use their vouchers at the stalls 3 miles away in the city centre but rose when the fruit and vegetable van began its weekly visits to intervention streets.

**Table 4:** Household participation and voucher distribution and redemption

**Site** Number of intervention streets	**Types of fruit and vegetable vendors** **(distance from streets)**	**Duration of exposure (weeks)**	**Weekly household participation rate**^**b**^	**Vouchers distributed/ vouchers redeemed**	**Average weekly voucher spend**	
					**Households receiving envelopes (*n* = 322)**	**All eligible households** **(*n* = 375)**
**Pilot RCT (2021–22)**						
**Tower Hamlets** 3	Four outdoor market stalls (~ 0.5 miles)	43	75% (73/97)	84% (£10,216/£12,170)	£3.26	£2.44
**Bradford** 3	Two indoor market stalls (~ 3 miles), One local community centre shop (~ 0.2 miles), One van weekly visits to streets	40	83% (104/125)	67% (£11,730/£17,440)	£2.82	£2.35
**Doncaster** 3	Two indoor market stalls (~ 2.5 miles)	19	95% (145/153) ^c^	54% (£4057/£7535)	£1.47	£1.39
**Previous feasibility studies (2017–2018)**						
**Sheffield** 1^a^	Two indoor market stalls (3 miles) & weekly veg bag delivery to flats	56	79% (41/52)	97% (£10,641/£11,000)	£4.63	£3.65
**Barnsley** 4	Three indoor market stalls (3 miles) or local shop (~ 0.3miles)	52	82% (80/97)	88% (£17,575/£19,982)	£4.22	£3.48

^a^One block of flats

^b^Households receiving weekly envelopes when notified that the scheme was ending relative to households offered envelopes at Week 1

^c^No checkpoint implemented in Doncaster thus some households receiving weekly envelopes may have not used any of the vouchers

#### Key stakeholder perspectives

Households reported increased access to healthier food options and a desire to continue a healthier lifestyle after the voucher scheme stopped. Some households shared their vouchers with other households, usually relatives.

Most (10/13) independent fruit and vegetable vendors approached agreed to accept the vouchers, but it was difficult to find local vendors i.e. 0.5 miles away or less. Although we managed to find vendors close to the streets in Tower Hamlets and Bradford, the Doncaster vendors were situated ~ 2.5 miles away in the city centre. The fruit and vegetable vendors found the voucher scanning app easy to use and reported new customers and existing customers buying more. Households and mystery shoppers reported that vendors were welcoming to customers with the vouchers and provided a good variety and quality of fruit and vegetables. Mystery shoppers found no evidence of vendors exchanging non-fruit and vegetable for the vouchers.

In total £36,238 worth of vouchers were scanned by the ten fruit and vegetable vendors. The largest amount was the Bradford mobile van which took £10,819 (92% of all vouchers redeemed in Bradford). The smallest amount was taken by a Tower Hamlets outdoor stall holder (£307) who dropped out early due to cash flow problems with the 6-week vendor payment cycle. Conversations with local public health teams highlighted the positive impact of the intervention in addressing the need for access to healthy, affordable food in low-income communities.

The goal of securing intervention funding for at least two years for the sample size required for the main trial was not met. Although two of the three local government public health teams were able to fully fund the vouchers (face value and printing costs) for their households for the two-year intervention, it was not possible to find all the resources required to operate the vendor payment system and to pack and deliver weekly envelopes. Following review of the interim pilot study results the research funder opted to close the study before the pilot study was completed. This was due to suboptimal household survey response rates, insufficient intervention funds for the planned main trial, and delays to the research.

## Discussion

### Main findings of this study

The intervention was developed and delivered in three different inner-city areas. This multi-component scheme (weekly doorstep delivered paper vouchers, recipes, and simple brief nutritional information) was well received by households and local public health teams. The baseline household survey response rate was lower than the 50% target for progression to the main trial.

Most local fruit and vegetable vendors approached accepted the vouchers. Three quarters or more of households regularly accepted the envelopes. Higher voucher use levels were seen when vendors were close by and in sites with longer scheme duration.

### What is already known on this topic?

In the UK, there is evidence that local vendor specific paper vouchers for fruit and vegetables targeted at low-income vulnerable young families with children under five are feasible [32]. However, the acceptability of interventions that target participants based on personal circumstances is sub-optimal [14]. Earlier feasibility tests of the Fresh Street place-based approach in two areas of high deprivation reported high levels of acceptability by households with local fruit and vegetable vendors reporting new customers and existing customers buying more [18, 19].

### What this study adds

This first pilot randomised controlled study provides additional evidence of acceptability of the intervention in three inner city areas of high deprivation. The baseline self-reported fruit and vegetable portions per day (range 3.7 to 4.2) were similar to the UK average of 3.7 [33] but below the UK government ‘5 a day’ recommendation.

Voucher uptake was lower when vendors were further away (as in Doncaster) and higher when vendors were close by (Tower Hamlets and Bradford).

Compared to previous tests [18, 19] household participation levels were similar (75–95% of households used the vouchers) (Table 4) but average weekly spend was lower.

Levels of household participation were higher than for ‘Healthy Start’ [14] e.g. Tower Hamlets 75% vs 59%, Bradford 83% vs 66%. This may in part be due to targeting areas with need (not individuals), making it easy for households to receive vouchers (via doorstep delivery), and removing the effort and stigma associated with schemes which require individuals to prove need [15, 16].

If the local fruit and vegetable vendor (Bradford van) was not available, or no local fruit and vegetable vendor was found (Doncaster), this meant households had to find the time and money to use public transport. Future studies should explore ways to support and evaluate more local fruit and vegetable outlets in food desert areas of high deprivation e.g. ‘pop up’ stalls [34] and mobile vans [35].

Vendors reported an increase in trade. Increased demand has the potential to provide a degree of market stability for local fruit and vegetable vendors, which is necessary for a diversified healthy food landscape [32]. Using local fruit and vegetable vendors rather than supermarkets helps keep financial expenditure on fruit and vegetables in the local area and supports local retail. This is important for addressing availability constraints that households face and to mitigate against the food deserts that exist within the current UK food system [36, 37].

The newly developed system for securely printing and redeeming the vouchers enabled fruit and vegetable vendors to scan the paper vouchers using a mobile-friendly app. This system worked well, is scalable and is now being used in multiple sites e.g. Fresh Street Community [34].

However, the 6-week system for reimbursing vendors was expensive to operate and too slow for those small-scale vendors who worked on short (24 h) cash cycles, and the systems for the weekly packing and delivery of envelopes to households were resource intensive. The Fresh Street Community study is exploring ways to increase the impact of the intervention by setting up fruit and vegetable stalls in community centres and community gardens [34, 38, 39] and reducing costs by households collecting vouchers from their local community centre.

### Limitations of the study

This study is limited by the small sample size and short duration. It is possible that there was some intervention spillover to control streets as some households shared vouchers. The baseline survey response rate was 47% but the actual response rate is likely to be lower due to the exclusion of a few streets with the lowest response rates prior to randomisation. Other household surveys encounter low response rates, e.g.22% in the 2023 ONS Annual Living Costs and Food Survey. Future trials should consider alternative methods of assessing the impact on households.

The study experienced challenges in obtaining funding for intervention costs as well as operational and logistical challenges and delays in approvals exacerbated by the impact of the COVID-19 pandemic on the health research system [40]. These factors contributed to funding for the study being discontinued which curtailed the planned in-depth process evaluation to understand how the intervention was experienced by key stakeholders.

## Supplementary Information

Supplementary Material 1.

Supplementary Material 2.

Supplementary Material 3.

Supplementary Material 4.

## Data Availability

Data is provided within the manuscript and supplementary information files.
