# Supplementary figures and images for: Place-based household vouchers for locally supplied fruit and vegetables: the Fresh Street pilot cluster randomised controlled trial
Source: BMC Public Health. 2025 Jan 3;25:29. doi: 10.1186/s12889-024-21062-y (PMC11697849; doi:10.1186/s12889-024-21062-y)

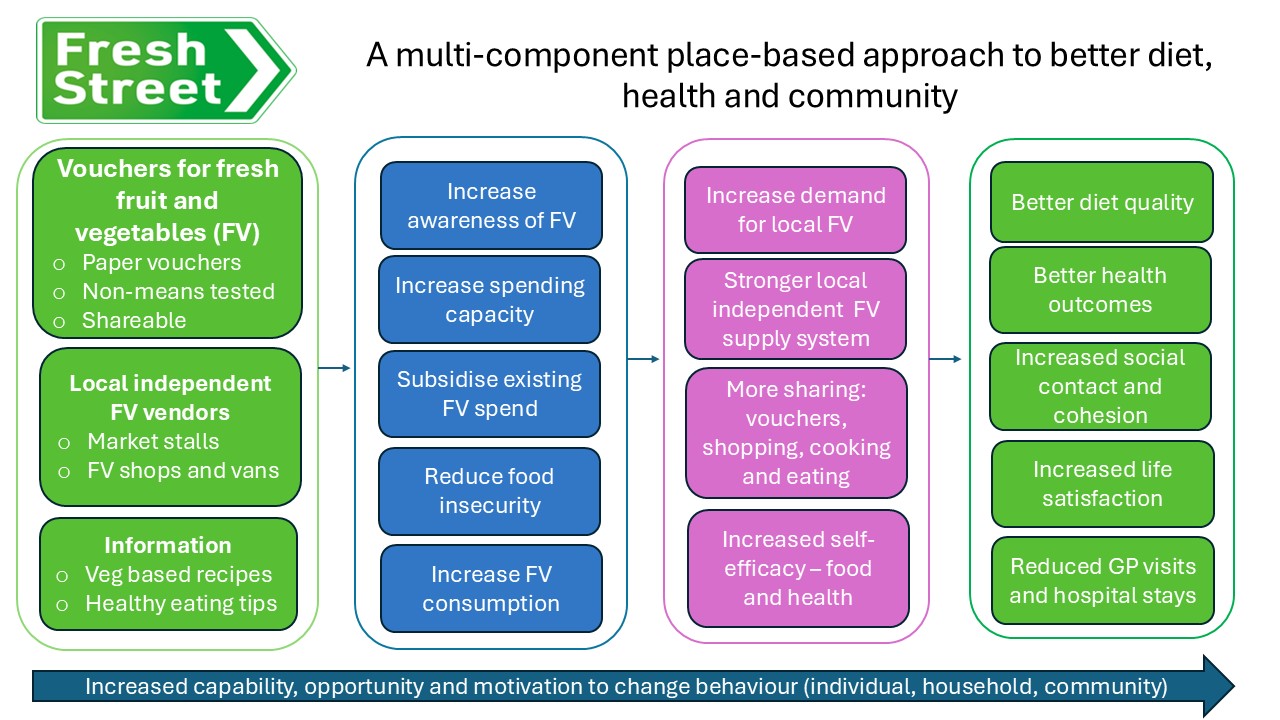

Supplement: Supplementary file 1 — Supplementary Material 1. [file 12889_2024_21062_MOESM1_ESM.jpg]
