# Supplementary material for: Place-based household vouchers for locally supplied fruit and vegetables: the Fresh Street pilot cluster randomised controlled trial
Source: BMC Public Health. 2025 Jan 3;25:29. doi: 10.1186/s12889-024-21062-y (PMC11697849; doi:10.1186/s12889-024-21062-y)
Supplement: Supplementary file 2 — Supplementary Material 2. [file 12889_2024_21062_MOESM2_ESM.pdf]

[SITE] Food & Health Survey

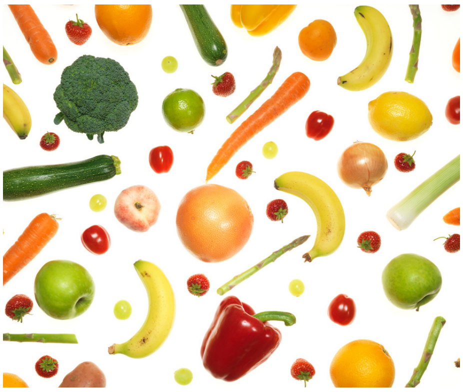

We all need food!
[SITE] are working with researchers to make sure that everybody has enough good **food** for a **healthy life.**

Please help us by answering a few questions
(and we'll give you a £5 shopping voucher to say thank you)

It should only take a few minutes to complete. All of the questions are optional. Any information you provide will only be used for research purposes.

Date survey completed: ....

**1: Where do you live?**			
House number:	....	Street name:	....
Please tick this box if you would like us to send you a supermarket voucher to ***thank you for filling in our survey.***	☐		

**2: How many portions of *FRUIT* did you eat yesterday?**		
Please include all fruit, including fresh, frozen, dried or tinned fruit, stewed fruit or fruit juices and smoothies.	....	portions

**3: How many portions of *VEGETABLES* did you eat yesterday?**		
Please include fresh, frozen, raw or tinned vegetables, but do not include any potatoes you ate.	....	portions

See enclosed information about portion sizes

**4: In the past month, how often have you eaten each of the foods below?**	*Never or occasionally*	*1-3 times a week*	*4-6 times a week*	*Daily*	*More than once a day*
Bacon, ham, sausages or burgers	☐	☐	☐	☐	☐
Coated or fried chicken	☐	☐	☐	☐	☐
Oily fish (sardines, salmon, mackerel, herring)	☐	☐	☐	☐	☐
White bread / pittas / naans etc.	☐	☐	☐	☐	☐
Wholemeal bread/ pittas / naans etc.	☐	☐	☐	☐	☐
Chips	☐	☐	☐	☐	☐
Salad or raw vegetables	☐	☐	☐	☐	☐
Crisps or savoury snacks	☐	☐	☐	☐	☐
Biscuits, cakes or pastries	☐	☐	☐	☐	☐
Sugary drinks (fizzy pop, squash)	☐	☐	☐	☐	☐
Beer, lager or cider	☐	☐	☐	☐	☐

5: Your health and wellbeing

Do you currently have any of the following symptoms? *(tick all that apply)*

- ☐ Fever *(temperature over 37.8c degrees)*
- ☐ Shortness of breath
- ☐ Muscle pain
- ☐ Persistent cough
- ☐ Sore throat
- ☐ Headache
- ☐ Runny nose
- ☐ Sneezing
- ☐ Nausea
- ☐ Blocked nose
- ☐ Wheezing
- ☐ Fatigue
- ☐ Loss or change to your sense of smell or taste
- ☐ None of these

Do you have any long-standing illness, health problem, condition or disability?
(tick all that apply)

- ☐ Tiredness/fatigue
- ☐ Pain
- ☐ Insomnia
- ☐ Anxiety/nerves
- ☐ Depression
- ☐ Memory problems
- ☐ Diabetes
- ☐ High blood pressure
- ☐ Heart disease
- ☐ Long COVID
- ☐ Breathing problems *(e.g. chronic bronchitis, asthma or emphysema)*
- ☐ Osteoarthritis
- ☐ Stroke
- ☐ Cancer
- ☐ None of these
- ☐ Other *(please state)*: ....

In the last THREE MONTHS, have you visited, consulted or received help from any of the following? ***(tick all that apply)***

- ☐ Hospital (in patient)
- ☐ Hospital (out patient)
- ☐ Hospital (day care)
- ☐ Accident and Emergency (A&E)
- ☐ Nurse
- ☐ Midwife
- ☐ GP/ family doctor
- ☐ Care worker
- ☐ Social worker
- ☐ Health Visitor /community nurse
- ☐ Counsellor
- ☐ None of the above
- ☐ Other *(please state)*: ....

GENERAL HEALTH: In general, would you say your health is:

☐ Excellent ☐ Very Good ☐ Good ☐ Fair ☐ Poor

6: About you	Male	Female	Other
Your gender	☐	☐	☐

Which one of the following best describes your ethnic group or background? *(please tick one box only)*

☐ *White British*	☐ *Other White background*	☐ *Mixed*	☐ *Asian or Asian British*
☐ *Chinese*	☐ *Black or Black British*	☐ *Other Ethnic Group*	☐ *Prefer not to say*

Your age *(years)*	☐ *18-24*	☐ *25-34*	☐ *35-44*	☐ *45-54*	☐ *55-64*	☐ *65-74*	☐ *75 or over*

Including yourself, how many people live in your household?	
How many are 4 years old or under?	
How many are aged between 5 and 18?	
How many are aged over 65?	

Can we contact you again?	Please give us any additional contact details:	
Yes ☐ *No* ☐		....<br>..<br>..

Thank you for taking part in the [SITE] Food & Health Survey. Your answers (combined with thousands of others) will help us find ways to keep us all healthy.

Please return this survey in the envelope provided to:

[SITE ADDRESS]

for office use

This survey is part of an independent research project led by Queen Mary University of London in collaboration with [SITE PARTNERS], University of Sheffield, University of Cambridge, and funded by the National Institute for Health Research (NIHR).

Queen Mary University of London | UNIVERSITY OF CAMBRIDGE | FUNDED BY NIHR National Institute for Health Research | The University Of Sheffield. | SITE LOGO PLACEHOLDER | SITE LOGO PLACEHOLDER
