# Supplementary material for: Place-based household vouchers for locally supplied fruit and vegetables: the Fresh Street pilot cluster randomised controlled trial
Source: BMC Public Health. 2025 Jan 3;25:29. doi: 10.1186/s12889-024-21062-y (PMC11697849; doi:10.1186/s12889-024-21062-y)

| 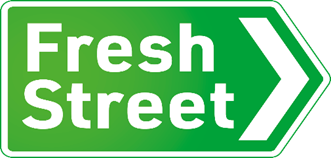 | Fresh Street Bradford  Phone: 01274435387  [hello@mylivingwell.co.uk](mailto:hello@mylivingwell.co.uk) |
| --- | --- |

17.03.2022

Hi,

**Here are this week’s vouchers.** This week’s recipe is great for batch-cooking, eat some now and use food containers to freeze the rest!

**See over for this week’s recipe: Winter Vegetable Soup**

You can spend your vouchers with any of these fruit and veg sellers:

**Solly’s Fruit and Veg Market Stall** *(Oastler Shopping Centre, John St, BD1 3SR).* Open: Monday to Saturday, 8am-6pm

**Sandale Community Hub and Shop** *(42-46 Reevy Road West, BD6 3LX)*

Open: Mon, Tues & Thurs, 8am to 3pm; Wed - 9am to 2pm; Sun - 10am to 2pm.

**Mobile Fruit and Veg Van.** Look out for Ash Hussain's van on Sundays at these times. Roy Road at 11am, Denham Drive at 12pm and Brafferton Arbour at 1pm. Ash will beep his horn to let you know he has arrived.

**Remember:** The **more** fruit and veg we eat (**especially veg**), the **healthier** we are and the **longer** we **live**. Five portions of fresh fruit and veg a day is good (but seven is even better!).

Best wishes

Jo, Michael, Jayne and Megan

*The Living Well service team in partnership with Fresh Street*

For more information see [www.freshstreet.uk](http://www.freshstreet.uk) or contact Fresh Street Bradford.

**Winter Vegetable Soup**

| **Ingredients**  200g red lentils  2 carrots  3 sticks celery  2 leeks  Tomato puree  4 garlic cloves  1 vegetable stock cube  Dried parsley and coriander  Seasonings (e.g., salt, pepper, chilli) | 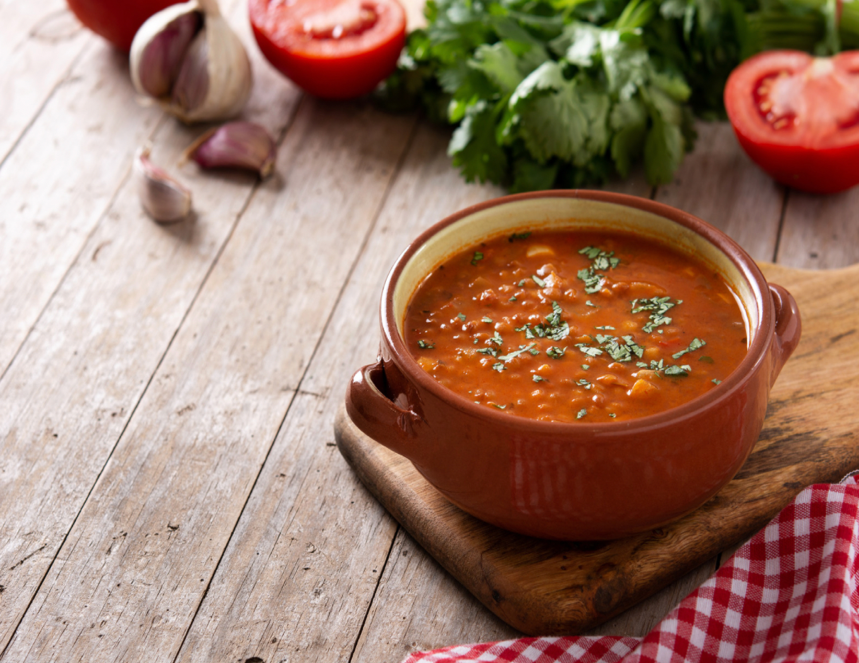 |
| --- | --- |
| *Servings: 4* | |
|  | |

**To Make**

Wash, cut and prepare all vegetables.

Tip all ingredients in large pan, pour over boiling water, then stir well.

Cover and leave to simmer for 30 minutes until vegetables and lentils are tender.

Pour into bowl to eat straightaway, or mash or blend one third if you like your soup to be ‘thick’.


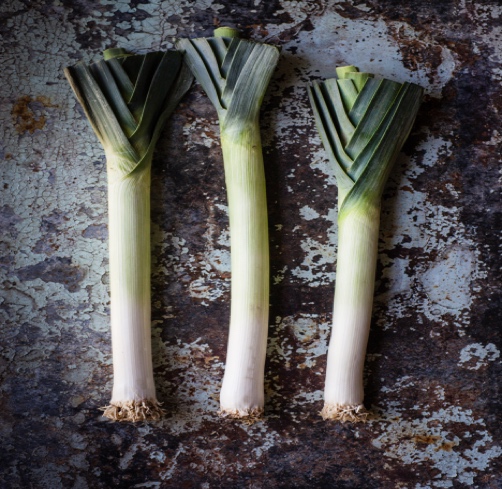
*Serve with toasted bread of your choice.*

***Did you know…****Leeks are rich in antioxidants and Vitamin K and will increase the strength of your immune and nervous system.*


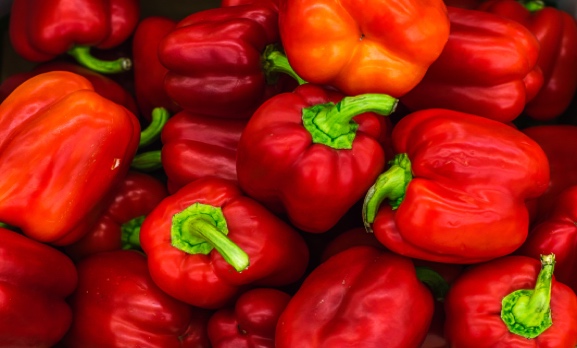

Supplement: Supplementary file 3 — Supplementary Material 3. [file 12889_2024_21062_MOESM3_ESM.docx]
