# Supplementary material for: Place-based household vouchers for locally supplied fruit and vegetables: the Fresh Street pilot cluster randomised controlled trial
Source: BMC Public Health. 2025 Jan 3;25:29. doi: 10.1186/s12889-024-21062-y (PMC11697849; doi:10.1186/s12889-024-21062-y)
Supplement: Supplementary file 4 — Supplementary Material 4. [file 12889_2024_21062_MOESM4_ESM.docx]

**Supplementary material 4: Proportion of households delivered to relative to number of households offered the intervention at week 1.**

* No delivery over Xmas but households received double vouchers the week before.

** Checkpoints: Envelope delivery ceased if no vouchers had been scanned by the checkpoint.

*** Households excluded in error rejoin. Error due to vendors not scanning redeemed vouchers in time.

| **Site** | **Tower Hamlets (N=97)** | **Bradford (N=125)** | **Doncaster (N=153)** |
| --- | --- | --- | --- |
| **Intervention week** | **(%)** | **(%)** | **(%)** |
| 1 | 98 | 100 | 100 |
| 2 | 94 | 98 | 98 |
| 3 | 91 | 96 | 98 |
| 4 | 91 | No delivery (Xmas)* | 98 |
| 5 | 92 | 93 | 98 |
| 6 | 90 | 92 | 97 |
| 7 | No delivery (Xmas)* | 90 | 97 |
| 8 | 92 | 90 | 97 |
| 9 | 78** Checkpoint | 90 | 97 |
| 10 | 78 | 69** Checkpoint | 97 |
| 11 | 78 | 79*** | 97 |
| 12 | 78 | 79 | 97 |
| 13 | 77 | 84 | 97 |
| 14 | 77 | 84 | 97 |
| 15 | 77 | 84 | 97 |
| 16 | 77 | 85 | 97 |
| 17 | 77 | 85 | 97 |
| 18 | 77 | 85 | 97 |
| 19 | 77 | 85 | 97 |
| 20 | 77 | 85 |  |
| 21 | 77 | 85 |  |
| 22 | 77 | 85 |  |
| 23 | 77 | 85 |  |
| 24 | 77 | 85 |  |
| 25 | 77 | 85 |  |
| 26 | 77** | 85 |  |
| 27 | 75 | 85 |  |
| 28 | 75 | 85 |  |
| 29 | 75 | 85 |  |
| 30 | 75 | 85 |  |
| 31 | 75 | 85 |  |
| 32 | 75 | 85 |  |
| 33 | 75 | 85 |  |
| 34 | 75 | 85 |  |
| 35 | 76 | 83 |  |
| 36 | 75 | 83 |  |
| 37 | 75 | 83 |  |
| 38 | 75 | 83 |  |
| 39 | 75 | 83 |  |
| 40 | 75 | 83 |  |
| 41 | 75 |  |  |
| 42 | 75 |  |  |
| 43 | 75 |  |  |
